# A Developmental Bias in Reading Frame Usage by Human Fetal Thymic TCRBDJ Transcripts is not Present in Genomic TCRBDJ Rearrangements

**DOI:** 10.1155/1999/16178

**Published:** 1999

**Authors:** James F. George, Yasushi Matsuura, Jacquelyn A. Byrne, Eugene L. Liu, Denise R. Shaw, John F. Kearney

**Affiliations:** 1 Department of Surgery Division of Cardiothoracic Surgery Department of Medicine University of Alabama Birmingham United Kingdom; 2 Division of Developmental and Clinical Immunology University of Alabama Birmingham United Kingdom; 3 Division of Hematology and Oncology University of Alabama Birmingham United Kingdom; 4 Department of Surgery Rm. 739 ZRB University of Alabama at Birmingham Birmingham Alabama 35294-0007 United Kingdom

**Keywords:** T-cell receptor, gene rearrangement, reading-frame usage

## Abstract

We have previously reported that reading-frame usage and functional diversification is developmentally regulated, with virtually all TCRB DJ mRNA transcripts using a single reading frame at 8 weeks of gestational age, tapering to 50% by adult life. We used the polymerase chain reaction to create genomic libraries of DJ rearrangements in the TCRB locus from thymuses at 7.7, 10, and 16 weeks of gestational age, and from adult thymuses. Clones were randomly picked and sequenced to determine junctional sequences and reading-frame utilization. The resulting data address the hypothesis that cells bearing genomic joints in reading frame one are preferentially selected during fetal life. This hypothesis predicts that reading- frame bias would also be observed among genomic DJ joints. Instead, we observed random utilization of the three possible D-region reading frames among genomic D1s1 => J1s1 joints during fetal life. Similar results were obtained at 7.7 weeks of gestational age in a second thymus in which both RNA and DNA were simultaneously isolated and used to create libraries of TCRBDJ transcripts or rearrangements. We conclude that reading-frame utilization is random among genomic D1s1-JB1s1 rearrangements and that the preferential usage of a single reading frame among mRNA transcripts of TCRB DJ transcripts is the result of preferential transcription of genomic TCRB DJ joints in a single reading frame, or that TCRB DJ transcripts have a longer half-life than transcripts in reading frames two or three.


*Developmental Immunology*, 1999, Vol.7(1), pp. 9-15
Reprints available directly from the publisher
Photocopying permitted by license only

© 1999 OPA (Overseas Publishers Association)
N.V. Published by license under the
Harwood Academic Publishers imprint,
part of Gordon and Breach Publishing Group
Printed in Malaysia

### A Developmental Bias in Reading Frame Usage by Human Fetal Thymic TCRBDJ Transcripts is not Present in Genomic TCRBDJ Rearrangements

JAMES F. GEORGE[a*], YASUSHI MATSUURA[b], JACQUELYN A. BYRNE[b], EUGENE L. LIU[a], DENISE R. SHAW[c] and JOHN F. KEARNEY[b]

[a]*Department of Surgery, Division of Cardiothoracic Surgery and Department of Medicine,* [b]*Division of Developmental and Clinical Immunology and* [c]*Division of Hematology and Oncology, University of Alabama at Birmingham*

*(Received 24 march, 1998)*

We have previously reported that reading–frame usage and functional diversification is developmentally regulated, with virtually all TCRB DJ mRNA transcripts using a single reading frame at 8 weeks of gestational age, tapering to 50% by adult life. We used the polymerase chain reaction to create genomic libraries of DJ rearrangements in the TCRB locus from thymuses at 7.7, 10, and 16 weeks of gestational age, and from adult thymuses. Clones were randomly picked and sequenced to determine junctional sequences and reading–frame utilization. The resulting data address the hypothesis that cells bearing genomic joints in reading frame one are preferentially selected during fetal life. This hypothesis predicts that reading–frame bias would also be observed among genomic DJ joints. Instead, we observed random utilization of the three possible D-region reading frames among genomic D1s1 => J1s1 joints during fetal life. Similar results were obtained at 7.7 weeks of gestational age in a second thymus in which both RNA and DNA were simultaneously isolated and used to create libraries of TCRBDJ transcripts or rearrangements. We conclude that reading–frame utilization is random among genomic D1s1-JB1s1 rearrangements and that the preferential usage of a single reading frame among mRNA transcripts of TCRB DJ transcripts is the result of preferential transcription of genomic TCRB DJ joints in a single reading frame, or that TCRB DJ transcripts have a longer half-life than transcripts in reading frames two or three.

*Keywords:* T-cell receptor, gene rearrangement, reading-frame usage

This work was supported by the National Institutes of Health, Grant R01 AI35940–01.

### INTRODUCTION

In previous studies, we established a system for examining the molecular events that occur during the rearrangement and expression of the TCRβ locus during early thymic development in humans. We found that the expression of partial rearrangements (D ==> J) in the human TCRβ locus during fetal life is regu-

* Address reprint requests and correspondence to James F. George, Ph.D., Department of Surgery, Rm. 739 ZRB, University of Alabama at Birmingham, Birmingham, Alabama 35294–0007, Phone: 205–934–4261 Fax: 205–934–5261, Email: jgeorge@uab.edu lated in a manner that suggests that there may be selective events that occur prior to the expression of a complete TCRβ chain on the cell surface. Of three possible reading frames, one D-region reading frame is predominantly used among DJ transcripts. At 8 weeks of gestation, 94% of the transcripts used reading frame one, a proportion that dwindled to 67% at 16 weeks and to 55% in the adult (George and Schroeder, 1992). Thus, the reading–frame bias is most pronounced during the critical 7–14 week period of gestation. Our hypothesis is that this bias in reading–frame utlization is a function of selection of thymocytes bearing rearrangements in reading frame one. Here we demonstrate that genomic TCRB D => J rearrangements exhibit random reading frame usage in contrast to D => J transcripts, which are highly biased in favor of a single reading–frame. Therefore, we conclude that the observed bias in reading–frame usage is not due to cellular selection, but is a result of either selective transcription or a longer half-life of DJ transcripts in reading frame one.

### RESULTS

### Structure and Composition of Genomic DB1s1-JB1s1 Rearrangements

A total of 127 unique genomic TCRB DJ clones were sequenced from thymuses obtained at gestational ages of 7.7 weeks (two thymuses, $n = 20$ and n=21), 10 weeks ($n$=24), 16 weeks ($n$=22), and adult life (two thymuses, $n$=22 and $n$=18). In accordance with our previous analysis of TCRBDJ transcripts (George and Schroeder, 1992), the developmental control of N-region addition also extends to genomic DJ joints. At 8 weeks of gestational age, the mean proportion of joints bearing N-region nucleotides was only 22%, but increased to 50% by 16 weeks of gestational age, reaching a maximum mean proportion of 83% during adult life (Table I). This increase in the proportion of genomic DJ joints bearing N-region nucleotides showed a positive linear correlation with gestational age through 16 weeks ($r = 0.98$: $Y = 3.35x - 2.91$). Similarly, the mean number of N-region nucleotides per genomic DJ joint also increased with time, with a positive linear correlation with increasing gestational age ($r = 0.99$: $Y = 0.27x - 0.93$). There were no differences in the average number of nucleotides removed from the genomic sequence of the gene segments composing the DJ joints (Table I).

### D-Region Reading–Frame Usage Is Random in Genomic DJ Joints

Figure 1 shows reading–frame usage among 106 unique DJ joints isolated from human thymuses obtained at gestational ages of 7.7, 10, 16 weeks, and from two thymuses obtained from healthy adults. Unlike fetal TCRB DJ mRNA transcripts, in which the reading-frame usage is strongly biased toward the use of a single reading–frame (George and Schroeder, 1992) genomic TCRB DJ joints exhibit random reading–frame usage during fetal life. Surprisingly, the proportion of genomic clones using reading frame one was 68.2% of the sequenced D => J gene segment joints in one adult thymus, which is significantly different from the theoretical stochastic value of 33% ($\chi$ square test, $p$= 0.05). However, no bias in reading frame usage was observed among genomic DJ joints isolated from a second adult thymus (Fig. 1). Therefore, we conclude that genomic TCRB DJ joints do not exhibit the strong bias in reading–frame usage that has been observed among TCRB DJ mRNA transcripts (George and Schroeder, 1992). To exclude the possibility that the observed characteristics of reading–frame usage among transcripts and genomic DJ joints was a result of the time and source of sample acquisition, we simultaneously isolated genomic DJβ rearrangements and DJβ transcripts from a second 7.7=week fetal thymus. Figure 2 shows that reading frame one was used in 19/23 mRNA transcripts (83%, $p = 0.01$ as compared to random utilization, $\chi$ square). In contrast, sequences isolated from genomic DNA obtained from the same tissue sample showed markedly random reading–frame usage with 38% of joints in reading frame one, 24% in reading frame 2, and 38% in reading frame three (Fig. 2). There was no statistically significant bias in the usage of reading frame one ($p = 1.0$, $\chi$ square) in the genomic DJ joints.

TABLE I Characteristics of Fetal TCRB DJ Genomic Rearrangements

| *Gestational age (weeks)* | *N* | *Mean[a] N-region length* | *Proportion bearing N-regions* | *Mean[b] deletion D-segment* | *Mean[b] deletion J-segment* | *Percent[c] germline D-segment* | *Percent[c] germline J-segment* |
|---|---|---|---|---|---|---|---|
| 7.7 Sample 1 | 20 | 1.2±0.4 | 25% | 2.9±2.2 | 3.0±2.2 | 20 | 0 |
| 7.7 Sample 2 | 21 | 1.3±0.5 | 19% | 2.4±2.3 | 3.3±2.8 | 29 | 19 |
| 10 | 24 | 1.6±0.7 | 33% | 3.0±2.6 | 3.5±2.9 | 21 | 8 |
| 16 | 22 | 3.5±3.9 | 50% | 3.2±2.3 | 3.1±2.0 | 18 | 5 |
| Adult Sample 1 | 22 | 5.0±3.6 | 77% | 4.6±2.8 | 3.0±3.0 | 5 | 14 |
| Adult Sample 2 | 16 | 5.5±2.4 | 88% | 3.2± 2.5 | 3.5±1.8 | 12 | 6 |

[a]Among isolates bearing N-region nucleotides.
[b]Number of nucleotides removed from the end of the genomic sequence of the gene segment.
[c]Percentage of isolates in which the entire genomic sequence of the gene segments is retained.

### DISCUSSION

The purpose of the present study was to characterize the patterns of rearrangement of genomic TCRB DJ joints during the most critical period of thymic development that immediately follows the influx of T–cell progenitors at 7 to 8 weeks of gestational age. DJ joints among partially rearranged TCRB genes were analyzed in order to sample the rearrangements prior to the period when full-length TCRB VDJ rearrangement and expression takes place, rendering thymocytes susceptible to T–cell–receptor–dependent positive and negative selection. We have previously analyzed the structure and composition of TCRB DJ mRNA transcripts isolated from fetal thymocytes at 8, 11, and 16 weeks of gestational age, and found a strong bias toward the usage of a single reading frame (George and Schroeder, 1992). The objective of the study in this report was to determine whether the bias observed among TCRB DJ transcripts also extends to genomic DJ rearrangements. The resulting data address the hypothesis that the usage of a single reading frame by DJ transcripts is a result of cellular selection of thymocytes bearing transcripts in reading frame one. This possibility predicts that reading–frame bias would also be observed among genomic DJ joints. Instead, we observed random utilization of the three possible D-region reading frames among genomic D1s1 => J1s1 joints during fetal life.

There are a number of nonexclusive mechanisms that could be invoked to account for the predominance of reading frame one observed among D => J transcripts in the T–cell–receptor β-chain locus. Each of these mechanisms have been observed to play a role in the generation of the reading–frame bias that has been observed in the immunoglobulin μ heavy–chain locus. In mouse B–cells, DJμ rearrangements using members of the Dfl16 and Dsp D-region families show a strong predominance in the usage of reading frame one that appears to be mediated by three mechanisms (Kaartinen and Makela, 1985; Ichihara et al., 1989; Gu et al., 1990). The first is the presence of sequence homologies in the diversity and joining gene segments that facilitate the splicing of gene segments at specific points and favor rearrangement in reading frame one (Feeney, 1992; Gu et al., 1990; Tornberg and Holmberg, 1995). The second mechanism is that the expression of gene products in reading frame three is prevented by stop codons. Lastly, there appears to be negative selection of cells expressing products in reading frame two that is dependent on external signaling through the immunoglobulin constant region. In mice in which the transmembrane portion of the Cμ constant region has been disrupted, reading–frame utilization is random (Gu et al., 1991). In addition, analysis of Abelson–virus–transformed cell lines has shown that DJμ transcripts in reading frame two are translated into a protein and expressed on the cell surface in association with surrogate light–chain proteins lambda 5 and Vpre B (Tsubata et al., 1991).

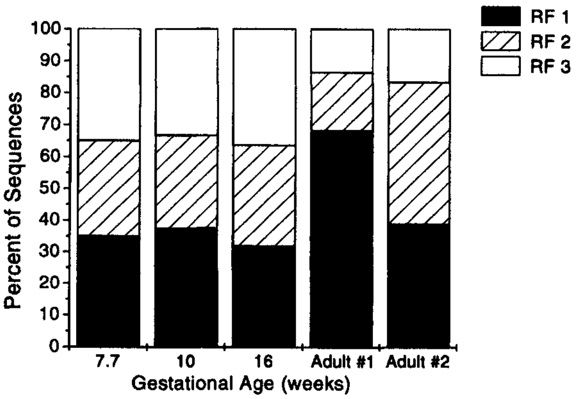

FIGURE 1 Reading–frame utilization in TCRB DJ sequences obtained from genomic DNA isolated from fetal and adult thymuses. The frequency of sequences wherein a Jβ gene segment has been spliced into D-region segment in RF 1 is depicted as a black column. Rearrangements in RF 2 are shown in cross–hatch and rearrangements in RF 3 are shown in white. The numerator in all cases is the total number of unique transcripts sequenced from the thymus sample defined on the x axis

It is unlikely that stop codons and sequence homologies contribute to the predominance observed in the TCRB locus because our data show that there are no sequence homologies between the D and J gene segments, and no stop codons in reading frame three. This leaves the possibilities of selective transcription or longer persistence of DJ transcripts in reading frame one.

In this analysis of genomic TCRBDJ rearrangements, it is clear that that the predominance of reading frame one among DJ transcripts from the T–cell–receptor β-chain locus can be attributed to mechanisms that are distinct from those seen in the reading–frame predominance observed in the immunoglobulin μ locus, and that the possible functional role of such transcripts may be different. As discussed before, major features of Igμ rearrangements that can be implicated in reading frame one predominance are not found in rearrangements in the TCRB locus, and full-length VDJμ rearrangements also exhibit a predominant reading frame, whereas full-length TCRB VDJ rearrangements show random reading–frame usage.

The functional role, if any, that TCRBDJ transcripts in reading frame one may play in T–cell development is unclear. At this time, we have found such transcripts in abundance in every fetal tissue that has been shown to participate in T–cell lymphopoiesis (unpublished data). Our studies are currently oriented toward the discovery of possible protein products of DJ transcripts and the isolation and characterization of cell subpopulations bearing TCRBDJ transcripts and germline TCRBDJ rearrangements in reading frame one.

### MATERIALS AND METHODS

### Genomic DNA Isolation and PCR Amplifications

Fetal thymuses of 8, 11, and 16 weeks of gestational age were obtained from the Department of Human Genetics at the University of Washington. Adult thymus tissue was obtained from normal adults during cardiac surgery. Genomic DNA was purified from these tissues as previously described (Ausubel et al., 1995).

One microgram of DNA was placed in a PCR reaction mix containing a sense oligonucleotide identical to the sequence located in the intron flanking the 5′ end of DB 1s1 (TGGTGGTCTCTCCCAGGCTCT) and an anti-sense oligonucleotide complementary to the sequence located in the intron between J1s1 and J1s2 (GCCACTCTAAAAGGGACACTG). The reaction mixtures contained 400 nM of each oligonucleotide, 0.2 mM dNTP, 50 mM KCl, 1.5 mM MgC12, 20 mM Tris-HCl (pH 8.4), 2.5 U Taq polymerase (Gibco BRL) in a volume of 100 μl. Amplification conditions consisted of an initial denaturation at 94°C for 5 min followed by 30 cycles at 94°C for 1 min, 55°C for 1 min, and 72°C for 2 min. A final 7–min extension was performed at 72°C. Amplified DNA corresponding to the TCRBDJ rearrangements was purified by agarose gel electrophoresis. The resulting gel slice was melted, diluted 1:10, and 1 μl was used in a nested amplification using the same–sense oligonucleotide upstream of the D1s1 gene segment and an anti-sense oligonucleotide complementary to the JB 1s1 coding region (TACAACTGTGAGTCTGGTGCC). Amplification conditions were the same as the first, except that no initial denaturation step was performed.

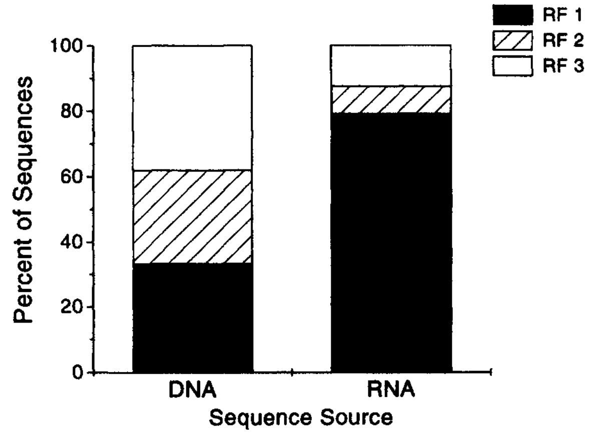

FIGURE 2 Reading–frame utilization in TCRB DJ transcripts (RNA) and genomic sequences (DNA) isolated from a fetal thymus of 7.7 weeks gestational age. The frequencies are depicted as indicated in Fig. 1

### RNA Isolation, cDNA Synthesis, and Amplification of TCRBDJ Transcripts

RNA was isolated using the acid phenol extraction method as described by Chomcynski and Sacchi (1987). First–strand oligo (dT) primed cDNA was generated from half of the RNA sample using recombinant reverse transcriptase (Superscript, Gibco/BRL, Gaithersburg, Maryland) in the buffer supplied by the manufacturer (Gubler and Hoffman, 1983). As a control, the remaining half of the isolated RNA sample was subjected to the same treatment in the absence of reverse transcriptase. Primers and amplification conditions were as previously described (George and Schroeder, 1992).

### Cloning of Amplified DNA and DNA Sequencing

Amplified DNA was purified by ethanol precipitation and subjected to agarose gel electrophoresis. The DNA fragment corresponding to the expected size was purified and ligated into pUC19 by TA cloning (Marchuk et al., 1991). This ligated plasmid was transfected into XL- 1 blue cells by electroporation (Gene Pulser, BioRad, Richmond, California). Randomly selected clones were isolated and sequenced using 7-deaza-dGTP analogs and sequenase version 2 (United States Biochemical, Cleveland, Ohio).

### Data Analysis

For the analysis of transcripts, we used previously described criteria for D-region gene assignment (George and Schroeder, 1992). Briefly, if a sequence has rearranged to a J1sX, we arbitrarily assume that the D, if present, is D1s1. If a sequence exhibits greater than or equal to 4 bp of sequence homology with a given D-region exon, assignment is made on that basis. In cases where there were less than four bases of identity with either D segment, the joining segment is Jβ1sX, and there are at least two bases of identity with D1s1, we arbitrarily assign this as D1s1.

Statistical analyses were performed using the student t-test, $\chi$ square, and linear regression analyses.

DEVELOPMENTAL IMMUNOLOGY
LOG
*DATE:* July 17 The next log will be mailed on August 3

| *Ms No.* | *Author* | *Ed. Recd* | *Ed. Accept* | *PMP Reod* | *To Type.* | *Status* |
|---|---|---|---|---|---|---|
| **Note: The papers for the Germinal Center Conference have been listed together and are now found at the end of the Log.** | | | | | | |
| | | **1996** | | | | |
| **Only the open papers for 1996 are listed.** | | | | | | |
| LDP9603 | Rodrigues et al3 | 02/02/96 | 05/14/97 | 05/17 | 08/13 | Comp |
| MAR044 | Freitas et al. | 07/22/96 | 09/03/96 | | | Acc/Major |
| MAR045 | Panwala et al. | 09/15/96 | | | | Acc/Minor |
| | | **1997** | | | | |
| **Only the open papers for 1997 are listed.** | | | | | | |

| *Ms No.* | *Author* | *Ed. Recd* | *Ed. Accept* | *PMP Reod* | *To Type.* | *Status* |
|---|---|---|---|---|---|---|
| RS96-RS025MOD | Mehr et al. | 01/06/97 | 09/30/97 | 10/02 | 11/05 | Comp |
| RS9701 | Huguet et al., | 01/06/97 | 07/10/97 | 07/14 | 07/19 | Comp |
| RS9704 | Kohen et al. | 03/05/97 | 07/24/97 | 07/28 | 08/13 | Comp |
| RS9705 | Reza & Ritter | 04/15/97 | 05/05/97 | 05/13 | 05/17 | Comp |
| RS9706 | Yang-Snyder & Rothenberg | 05/30/97 | 06/21/97 | 07/14 | 07/19 | Comp |
| MAR048 | Reya et al. | 06/12/97 | 08/22/97 | 09/05 | 11/05 | Comp |
| LDP9702 | Horton et al. | 08/24/97 | 01/23/98 | 01/30/98 | 02/23/98 | Comp |
| MAR049 | Vicente et al. | 08/22/97 | 0209/98 | 02/16/98 | 02/23/98 | Comp |
| | | **1998** | | | | |
| RS9801 | Freysdottir & Ritter | 01/05/98 | | | | Review |
| LDP9801 | Enomoto & Tochinai | 02/27/98 | | | | Review |
| **LDP9802** | **George et al.** | **03/29/98** | **05/05/98** | **05/09/98** | **07/18/98** | **Comp** |
| RS98–02 | Athanassakis et al. | 05/20/98 | | | | Review |
| RS9803 | Masayoshi ??? et al. | 05/21/98 | | | | Review |

OVER TO NEXT PAGE FOR GCC PAPERS AND NOTES

**PMP EDITORIAL & PRODUCTION**
**3 Four Corners Road/Warwick, NY 10990–3014**
**Phone: 914–986–5750; Fax: 914–986–6119; e-mail: pjzurita@warwick.net**

**Germinal Centre Conference Papers**

| | | | | | | |
|---|---|---|---|---|---|---|
| RB9601 | Rizzo et al. | 08/20/96 | 05/09/97 | 08/23 | 11/05 | Comp* |
| RB9602 | Nahn et al. | 08/10/96 | | | | Acc/Major* |
| RB9603 | Rosenberg et al. | 09/02/96 | 05/30/97 | 07/25 | 08/13 | Comp* |
| RB9604 | Suzuki et al. | 09/04/96 | | | | Acc/Major* |
| RB9605 | Colic et al. | 08/05/96 | 08/10/97 | 08/23/97 | 09/27/97 | Comp* |
| RB9606 | Cukrowska et al. | 08/12/96 | 08/19/97 | 08/23/97 | 09/27/97 | Comp* |
| RB9607 | Brandtzaeg & Far. | ??? | | | | Acc/Major* |
| RB9608 | De Waard et al. | ??? | 05/30/97 | 07/25 | 08/13 | Comp* |
| RB9609 | Forster | ??? | 05/3097 | 07/25 | 08/13 | Comp* |
| RB9610 | Gärdby et al. | 08/11/96 | 08/14/97 | 08/23/97 | 09/27/97 | Comp* |
| RB9611 | Gail et al. | ??? | 05/30/97 | 07/25 | 08/13 | Comp* |
| RB9612 | Hunt et al. | ??? | 05/30/97 | 07/25 | 08/13 | Comp* |
| RB9613 | Krenac & Rosendaal | ??? | 05/30/97 | 07/25 | 08/13 | Comp* |
| RB9614 | Lukiuc et al. | ??? | 05/30/97 | 07/25 | 08/13 | Comp* |
| RB9615 | Marx et al. | ??? | 05/30/97 | 07/25 | 08/14 | Comp* |
| RB9617 | Specht et al. | ??? | | 09/29 | 11/05 | Comp* |
| RB9618 | Varas et al. | ??? | 05/30/97 | 07/25 | 08/13 | Comp* |
| RS9707 | Classon & Boyd | 06/16/97 | 06/19/97 | 06/23 | 06/30 | Comp* |
| KS9601 | Arnoldi & Moll | 08/13/96 | 08/18/97 | 08/23 | 09/27 | Comp* |
| KS9602 | Balogh et al. | 08/06/96 | 08/12/97 | 08/23 | 10/4 | Comp* |
| KS9603 | Cebra et al. | 08/16/96 | 08/14/97 | 08/23 | 09/27 | Comp* |
| KS9605 | Chidgey et al. | 01/20/97 | 08/19/97 | 08/23 | 09/27 | Comp* |
| KS9606 | Eichmann | 09/15/96 | 05/09/97 | 08/23 | 09127 | Comp* |
| KS9607 | Gieseler et at. | 08/15/96 | 08/12/97 | 08/23 | 09/27 | Comp* |
| KS9608 | Greiner et al. | 10/12/96 | 05/09/97 | 08/23 | 10/4 | Comp* |
| KS9609 | Hutter et al. | 10/21/96 | 05/09/97 | 08/23 | 10/4 | Comp* |
| KS9610 | Imami et al. | 10/20/96 | 05/09/97 | 08/23 | 10/4 | Comp* |
| KS9611 | Kadmon et al. | 08/15/96 | 08/10/97 | 08/23 | 10/4 | Comp* |
| KS9612 | Laman et al. | 11/06/96 | 08/12/97 | 08/23 | 10/4 | Comp* |
| KS9613 | Nieuwenhuis | 08/20/96 | 08/18/97 | 08/23 | 09/27 | Comp* |
| KS9614 | Press et al. | 08/06/96 | 08/18/97 | 08/23 | 10/28 | Comp* |

| *Ms No.* | *Author* | *Ed. Recd* | *Ed. Accept* | *PMP Reod* | *To Type.* | *Status* |
|---|---|---|---|---|---|---|
| KS9615 | Ramirez | 08/15196 | 08/18/97 | 08/23 | 10/28 | Comp* |
| KS9616 | Rinner et al. | 10/01/96 | 08/18/97 | 08/23 | 10/28 | Comp* |
| KS9618 | Ryffel et al. | 08/05/96 | 08/10/97 | 08/23 | 10/28 | Comp* |
| KS9619 | Schilizzi et al. | 08/16/96 | 08/12/97 | 08/23 | 10/28 | Comp* |
| KS9620 | Skibinski et al.. | 08/05/96 | 08/14/97 | 0823 | 11/05 | Comp* |
| KS9621 | Smith et al. | 11/02/96 | 08/18/97 | 08/23 | 11/05 | Comp* |
| KS9622 | Thellin et al. | 08/07/96 | 08/10/97 | 08/23 | 11/05 | Comp* |
| KS9623 | Vora et al. | 09/10/96 | 05/09/97 | 08/23 | 11/05 | Comp* |
| KS9624 | de Mello-Coelho et al. | 08/15/96 | 08/10/97 | 08/23 | 11/05 | Comp* |

Note: Lines in red are those that have changed since the last report.
Boldface S after "To Type." Date is the scheduled date.
Acc/Major = Accepted with major revision.
Acc/Minor = Accepted with minor revision.
Asterisk (*) = Paper for GCC Special Issue.
Copies to : S. D. Hewitt; Andrea Walcott; Roland Scollay; John F. Kearney; Christopher C. Goodnow; Tadamitsu Kishimoto; Louis Du Pasquier; Mary A. Ritter
imulog

### *Acknowledgements*

We would like to thank Cathy L. Jones and Ann Elizabeth Ratcliffe for excellent technical assistance. We also thank Dr. Harry W. Schroeder, Jr. for helpful discussions.

### *References*

Ausubel, F.M., Brent, R., Kingston, R.E., Moore, D.D., Seidman, J.G., Smith, J.A., and Struhl, K. (1998). Current protocols in molecular biology (New York: John Wiley).

Chomcynski, P., and Sacchi, N. (1987). Single-step method of RNA isolation by acid guanidinium thiocyanate-phenol-chloroform extraction. *Anal. Biochem* **162:** 156–159.

Feeney, A.J. (1992). Predominance of $V_H$-D-$J_H$ junctions occurring at sites of short sequence homology results in limited junctional diversity in neonatal antibodies. *J. Immunol.* **149:** 222–229.

George, J.F., and Schroeder, H.W. (1992). Developmental regulation of Dβ reading frame and junctional diversity in T cell receptor-β transcripts from human thymus. *J. Immunol.* **148:** 1230–1239.

Gu, H., Forster, I. and Rajewsky, K. (1990). Sequence homologies, N sequence insertion and JH gene utilization in $V_HDJ_H$ joining: Implications for the joining mechanism and the ontogenetic timing of Ly1 B cell and B-CLL progenitor generation. *EMBO J.* **9:** 2133–2140.

Gu, H., Kitamura, D., and Rajewsky, K. (1991). B cell development regulated by gene rearrangement: Arrest of maturation by membrane-bound Dmu protein and selection of $D_H$ element reading frames. *Cell* **65:** 47–54.

Gubler, U., and Hoffman, B.J. (1983). A simple and very efficient method for generating cDNA libraries. *Gene* **25:** 263–269.

Ichihara, Y., Hayashida, H., Miyazaw, S., and Kurosawa, Y. (1989). Only DFL16, DSP2 and DQ52 gene families exist in mouse immunoglobulin heavy chain diversity gene loci, of which DFL16 and DSP originate from the same primordial $D_H$ gene. *Eur. J. Immunol.* **19:** 1847–1854.

Kaartinen, M., and Makela, O. (1985). Reading of D-genes in variable frames as a source of antibody diversity. *Immunol. Today* **6:** 117–185.

Marchuk, D., Drumm, M., Saulino, A., and Collins, F.S. (1991). Construction of T–vectors, a rapid and general system for direct cloning of unmodified PCR products. *Nucleic Acids Res.* **19:** 1154–1154.

Tornberg, U.C., and Holmberg, D. (1995). B-1a, B-1b and B-2 B cells display unique VHDJH repertoires formed at different stages of ontogeny and under different selection pressures. *EMBO J.* **14**: 1680–1689.

Tsubata, T., Tsubata, R. and Reth, M. (1991). Cell surface expression of the short immunoglobulin μ-chain (Dμprotein) in murine pre-B cells is differently regulated from that of the intact mu chain. *Eur J. Immunol.* **21:** 1359–1363.

